# Liquid Platelet-Rich Fibrin and Heat-Coagulated Albumin Gel: Bioassays for TGF-β Activity

**DOI:** 10.3390/ma13163466

**Published:** 2020-08-06

**Authors:** Zahra Kargarpour, Jila Nasirzade, Layla Panahipour, Richard J. Miron, Reinhard Gruber

**Affiliations:** 1 Department of Oral Biology, Medical University of Vienna, 1090 Vienna, Austria; zahra.kargarpooresfahani@meduniwien.ac.at (Z.K.); jila.nasirzaderajiri@meduniwien.ac.at (J.N.); layla.panahipour@meduniwien.ac.at (L.P.); 2 Department of Periodontology, School of Dental Medicine, University of Bern, 3012 Bern, Switzerland; richard.miron@zmk.unibe.ch

**Keywords:** Albumin, fibrin, platelet-rich fibrin, platelet-poor plasma, TGF-β

## Abstract

Liquid platelet-rich fibrin (PRF) can be prepared by high centrifugation forces separating the blood into a platelet-poor plasma (PPP) layer and a cell-rich buffy coat layer, termed concentrated PRF (C-PRF). Heating the liquid PPP was recently introduced to prepare an albumin gel (Alb-gel) that is later mixed back with the concentrated liquid C-PRF to generate Alb-PRF. PRF is a rich source of TGF-β activity; however, the overall TGF-β activity in the PPP and the impact of heating the upper plasma layer remains unknown. Here, we investigated for the first time the in vitro TGF-β activity of all fractions of Alb-PRF. We report that exposure of oral fibroblasts with lysates of PPP and the buffy coat layer, but not with heated PPP, provoked a robust increase in the TGF-β target genes interleukin 11 and NADPH oxidase 4 by RT-PCR, and for IL11 by immunoassay. Consistent with the activation of TGF-β signaling, expression changes were blocked in the presence of the TGF-β receptor type I kinase inhibitor SB431542. Immunofluorescence and Western blot further confirmed that lysates of PPP and the buffy coat layer, but not heated PPP, induced the nuclear translocation of Smad2/3 and increased phosphorylation of Smad3. The immunoassay further revealed that PPP and particularly BC are rich in active TGF-β compared to heated PPP. These results strengthen the evidence that not only the cell-rich C-PRF but also PPP comprise a TGF-β activity that is, however, heat sensitive. It thus seems relevant to mix the heated PPP with the buffy coat C-PRF layer to regain TGF-β activity, as proposed during the preparation of Alb-PRF.

## 1. Introduction

Platelet-rich fibrin (PRF), originally introduced in 2006 in an article series [1], has received increasing attention in clinical regenerative dentistry [2] and other fields, including aesthetic medicine [3], as an autologous source of cells and growth factors embedded in a fibrin-rich extracellular matrix that has handling properties similar to a graft or biomaterial. Even though the preparation of solid PRF is simple, as it basically requires the spontaneous coagulation of centrifuged blood, the protocols have been refined towards gaining an ideal ratio of the PRF clot and its content of platelets, leucocytes, and consequently also growth factors. However, apart from swing-out rotors causing a superior separation of the phases than fixed angle rotors [4], it is the clotting time, the g-force and the centrifugation time that define the properties of the PRF, also reflected by the various acronyms, including leukocyte-rich L-PRF and advanced A-PRF [5], most of which are, however, linked to a certain brand of blood collection tubes and centrifuges [6].

Traditionally, solid PRF was introduced as L-PRF prepared with blood collection tubes containing clot activators at around 700 g for 12 min in a fixed angle centrifuge. L-PRF has been shown to increase the width of the keratinized mucosa around implants [7], improve refractory skin ulcers [8] and favor socket management and ridge preservation [9]. Considering that in L-PRF, platelets and leucocytes are restricted to the buffy coat, A-PRF (200 g) protocols showed a rather equal distribution of platelets in the PRF [10,11]. More recently, PRF protocols prepared via horizontal centrifugation using plain glass tubes without clot activators to prepare H-PRF have been shown to lead to more favorable accumulation of platelets and leukocytes in the upper plasma layers [4,12]. Nevertheless, L-PRF and H-PRF membranes undergo spontaneous fibrinolysis within a 3 week period upon subcutaneous implantation in immunocompromised rodents [13], and thus are not ideal for clinical application requiring long-term volume stability serving as dermal fillers [14,15] or as a replacement for soft tissue grafts in dentistry [16].

Fibrinolysis is caused by the rather heat labile plasmin that is stored in the clot as an inactive form [17]. Inactivation of plasmin by pasteurization is a critical issue in the dairy industry [18]. Moreover, blood plasma is a rich source of albumin, being sensitive to heat-induced coagulation [19]. These common principles of heat inactivation, together with the historical understanding of the accumulation of leucocytes and also platelets in the buffy coat after centrifugation, prompted Miron et al. to develop a dual strategy to prepare PRF. They used plastic tubes with a hydrophobic surface to delay coagulating following the L-PRF protocol, thereby generating a large yellow plasma fraction [11,20]. This yellow clot consists of an almost cell-free liquid platelet-poor plasma (PPP) and the small cell-rich buffy coat [4]. To block fibrinolysis and simultaneously to provoke albumin coagulation, the PPP is subjected to heated at 75 °C for 10 min. After cooling, the PPP gel is mixed back with the original buffy coat fraction that maintained the coagulating properties. The liquid buffy coat is also termed concentrated PRF or C-PRF [11]. Thus, the viscous injectable PRF mixture consists of a gel prepared from heated PPP and the non-heated liquid buffy coat C-PRF fraction (Alb-PRF). Following subcutaneous implantation in nude mice, Alb-PRF, but not traditional L-PRF and H-PRF, remains stable over at least 21 days, ideally supporting medical procedures which require a long-lasting scaffold [13].

Heating, however, not only inactivates plasminogen and coagulates albumin; it might also affect the activity of the catalase [21] and growth factors intrinsic to the PRF clot. We recently performed a proteomic analysis combined with a whole genome gene array of a traditional PRF membrane and identified that TGF-β was a major growth factor with respect to its activation of genes in oral fibroblasts [22]. TGF-β activity is identified by its ability to regulate respective target genes, most notably IL11 and NOX4, that appeared in multiple independent screening approaches involving enamel matrix proteins [23], bone conditioned medium [24] and acid bone lysates [25]. The impact of heating on the activity of TGF-β is biphasic–TGF-β, because of its binding to latent TGF-β binding proteins, gaining activity by heating [26], while temperatures above 90 °C result in thermal denaturation of TGF-β [27]. Considering that TGF-β is a pleiotropic growth factor that is critically involved in wound healing and bone regeneration [28], and since some of TGF-β effects in vivo are mediated via IL11 [29,30] and NOX4 [31], bioassays based on the expression of these two sensitive target genes are suitable to measure TGF-β activity in PRF.

The question which arises concerns whether untreated PPP, being almost devoid of platelets and leukocytes, holds a TGF-β activity. If yes, does the preparation of albumin gels by heating 75 °C for 10 min affect its activity [20]. Another question which arises is if the cell-rich buffy coat is superior to PPP in the activation of TGF-β signaling in oral fibroblasts.

## 2. Results

### 2.1. PPP and Buffy Coat Lysates but Not of Heated PPP Enhance TGF-β Target Gene Expression in Fibroblasts

**Figure 1:**
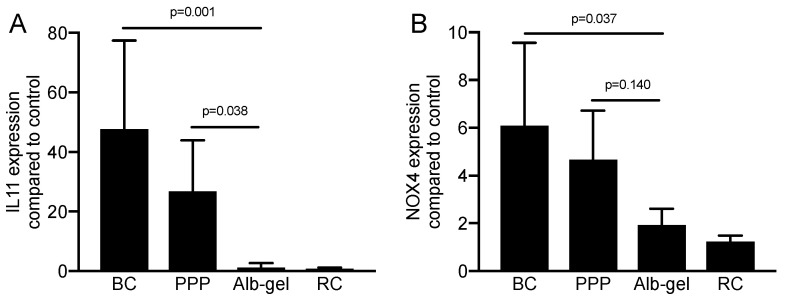
Platelet-poor plasma (PPP) and buffy coat but not heated PPP induced a substantial increase in TGF-ß target genes. Gingival fibroblasts were exposed to 30% of the respective lysates prepared from PPP, heated PPP (75 °C for 10 min; Alb-gel), and buffy coat (BC). For red clot (RC), 5% of the lysates was used. Expression analysis of (**A**) IL11 and (**B**) NOX4 was expressed as x-fold increases compared to the basal levels of untreated cells. Bar graphs show the means and standard deviation of four independent experiments (N = 4). Statistical analysis was based on an ANOVA with a multiple comparison against heated PPP based on Fischer’s LSD test.

To investigate the impact of blood fractionation in regulating the TGF-β activity of PRF, we performed a 700 g centrifugation for 8 min in hydrophobic plastic tubes [20]. This protocol allows a robust separation of the PPP from the red blood cells with the intermediate buffy coat layer. For preparing an Alb-gel, the PPP was heat denatured at 75 °C for 10 min followed by immediate incubation on ice [20]. We then analyzed the effects of the lysates prepared from PPP, heated PPP, buffy coat and red clot on the expression changes of the TGF-β target genes IL11 and NOX4 in human gingival fibroblasts. We observed the expected increase in the expression of IL11 and NOX4 when the fibroblasts were exposed to lysates of the buffy coat layer (Figure 1).

**Figure 2:**
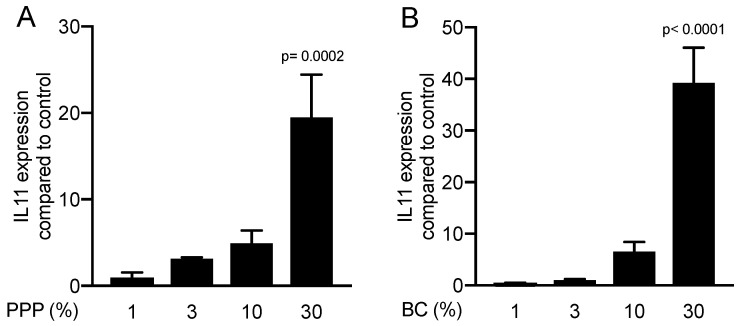
The effect of PPP and buffy coat is dose-dependent. Gene expression analysis for IL11 in gingival fibroblasts incubated with different concentrations of (**A**) PPP and (**B**) buffy coat (N = 3). Data are presented as mean ± SD.

Despite being almost devoid of platelets and other cells [10], lysates of PPP initiated a robust activation of IL11 and NOX4 gene expression. In contrast, however, lysates of heated PPP serving as control for statistics, or the red blood cell layers, failed to cause any expression changes (Figure 1). To identify whether there is a dose-dependent response in the presence of different concentrations of PPP and buffy coat, gingival fibroblasts were exposed to 1 to 30% of PPP and buffy coat. Only 30% of PPP and buffy coat was sufficient to induce a significant increase in IL11 (Figure 2).

### 2.2. PPP and Buffy Coat Lysates but not Heated PPP Caused the Accumulation of IL11 in the Cell Supernatant

**Figure 3:**
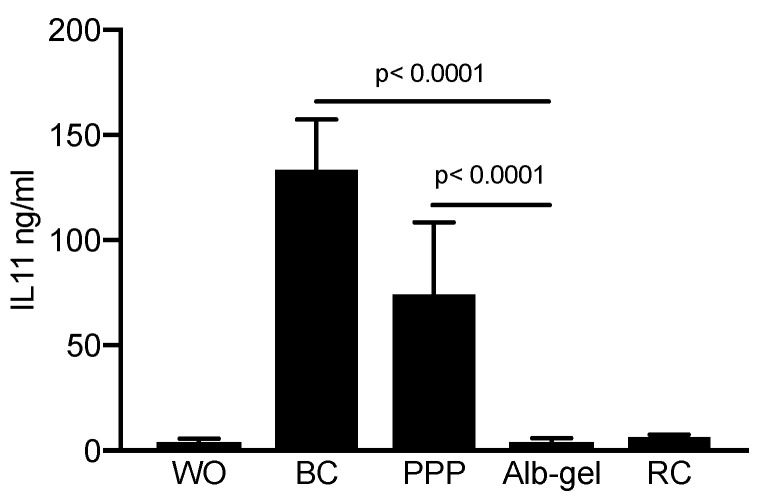
PPP and buffy coat but not heated PPP caused the accumulation of IL11 in the oral fibroblast cells supernatant. Gingival fibroblasts were exposed to 30% of the respectively lysates prepared from PPP, heated PPP (75 °C for 10 min; Alb-gel), and buffy coat (BC). For red clot, 5% of the lysates was used. The levels of IL11 in the supernatant of the fibroblasts is presented in ng/mL. Bar graphs show the means and standard deviation of three independent experiments (N = 3). Statistical analysis was based on an ANOVA with a multiple comparison against heated PPP based on Fischer’s LSD test.

To better support the findings obtained by gene expression analysis, we measured the protein levels of IL11 in the supernatant of the gingival fibroblasts. In line with the strong increase in IL11 transcripts, lysates of PPP and the buffy coat caused the accumulation of IL11 on the protein level by at least 20-fold compared to unstimulated controls. Consistently, heated PPP, which served as a control in the statistical analysis, and red blood cell lysates had no substantial impact on the production of IL11 by gingival fibroblasts (Figure 3).

### 2.3. Inhibition of the TGF-β Receptor Type I Kinase Blocked the Expression of Respective Target Genes

**Figure 4:**
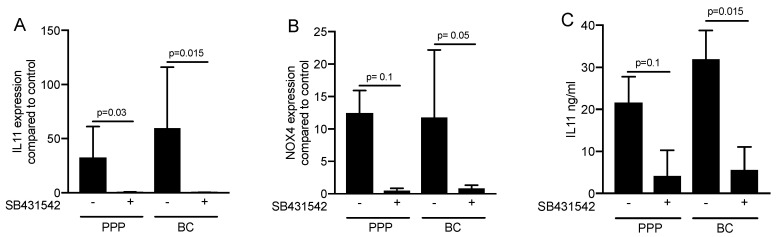
Inhibition of the TGF-β receptor type I kinase blocked the expression of respective target genes. Gingival fibroblasts were exposed to 30% of PPP and buffy coat (BC) with and without the TGF-β receptor 1 antagonist SB431542. Expression analysis of (**A**) IL11 and (**B**) NOX4 is expressed as x-fold increases compared to the basal levels of untreated cells. (**C**) The levels of IL11 in the supernatant of the fibroblasts was presented in ng/mL. Bar graphs show the means and standard deviation of four independent experiments (N = 4). Statistical analysis was based on a paired *t*-test.

To confirm the involvement of TGF-β signaling to regulate the respective genes, the TGF-β receptor 1 antagonist SB431542 was introduced [32]. We report that SB431542 blocked the activity of PPP and buffy coat lysates to increase the expression of IL11 and NOX4 (Figure 4A,B). Further support for the involvement of TGF-β signaling comes from the immunoassay, showing that SB431542 blocked the accumulation of IL11 in the supernatant of cells exposed to PPP and buffy coat lysates (Figure 4C). These observations support the notion that the effects of the PPP and buffy coat lysates are mediated via the TGF-β receptor.

### 2.4. Lysates of PPP and the Buffy Coat but not Heated PPP Induced an Activation of Smad2/3 Signaling

**Figure 5:**
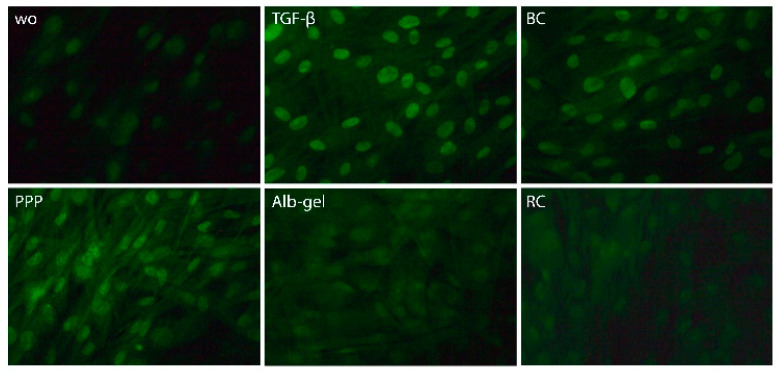
Lysates of PPP and the buffy coat layer but not heated PPP provoked nuclear translocation of Smad2/3. Gingival fibroblasts were exposed for 1 h to 30% of the lysates prepared from PPP, heated PPP (75 °C for 10 min, Alb-gel), and buffy coat (BC). For red clot, 5% of the lysates was used. Immunofluorescence revealed the nuclear translocation of Smad2/3 by lysates prepared from PPP and the buffy coat (BC) but not when cells were exposed to lysates of heated PPP and red clot.

In support of the activation of TGF-β receptor signaling [33], immunofluorescence and Western blot analysis were performed. Consistent with the proposed TGF-β activity, PPP lysates and buffy coat but not the heated PPP triggered the nuclear translocation of Smad2/3 in gingival fibroblasts (Figure 5).

**Figure 6:**
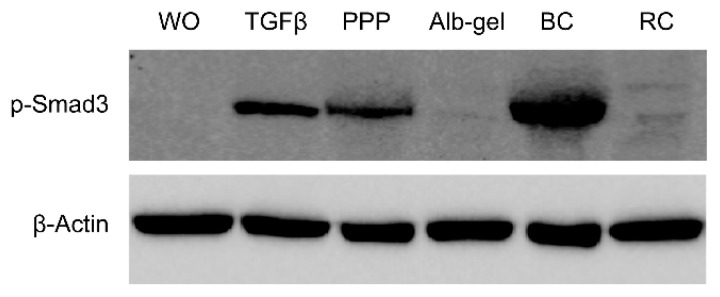
Lysates of PPP and the buffy coat layer but not heated PPP provoked phosphorylation of Smad3. Gingival fibroblasts were exposed for 1 h to 30% of the lysates prepared from PPP, heated PPP (Alb-gel) (75 °C for 10 min), and buffy coat (BC). For red clot (RC), 5% of the lysates was used. Western blot showed a strong increase in the basal Smad3 phosphorylation signal by lysates prepared from PPP and the buffy coat (BC) but not when cells are exposed to lysates of heated PPP and red clot.

Knowing that phosphorylation of Smad3 is a prerequisite for its nuclear translocation, it was demonstrated that both the PPP lysates and buffy coat C-PRF layer activated the increased phosphorylation of Smad3, while heated PPP and red blood lysates had no effects (Figure 6).

**Figure 7:**
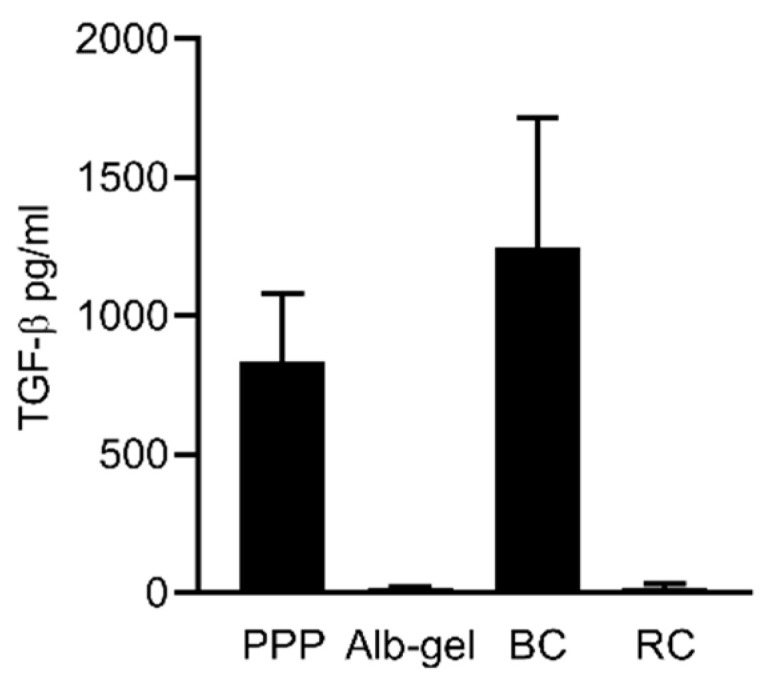
Concentration of active TGF-β in the all the fractions of PPP, Alb-gel, buffy coat (BC), and red clot (RC). Active TGF-β in all the prepared fractions was determined by immunoassay. Data represent the mean and standard deviation. TGF-β is indicated in pg/mL (N = 4–5).

To evaluate the concentration of TGF-β in all the fractions, a TGF-β immunoassay was conducted. The results show that the BC (~1200 pg/mL) and PPP (~900 pg/mL) layer has the highest concentrations of TGF-β1 compared to Alb-gel (~20 pg/mL) and RC (~20 pg/mL), respectively (Figure 7).

## 3. Discussion

This research was prompted by the recently established PRF formulation consisting of heat-coagulated PPP mixed with the liquid cell-rich buffy coat C-PRF to serve as a long-term stable autologous and injectable matrix [13,20]. Previous reports clearly demonstrated that TGF-β is among the growth factors supporting the formation of a fibrous extracellular matrix [29,30,34]. The overall goal of the work presented here was therefore to assess whether the cell-free PPP holds a TGF-β activity, and if yes, how the activity is affected by heating. We found that lysates of PPP, being almost devoid of platelets and other cells and, as expected, also the buffy coat layer, are potent sources of TGF-β activity. Conversely, lysates of heated PPP, similar to the red blood cell fraction, failed to provoke the expression of the TGF-β target genes and the activation of upstream signaling consisting of activation of the TGF-β receptor 1 kinase [32], as well as the phosphorylation and nuclear translocation of Smad2/3 [33]. This finding is in line with the catalase activity of PRF lysates that is abolished by heating [21]. There were variations among the blood and cell donors but the overall finding that PPP has a weaker TGF-β activity than buffy coat was consistent among all experiments. Taken together, these results are important because they support the proposed protocol to reconstitute the biological activity of the heat-coagulated PPP by the supplementation with a TGF-β-rich liquid buffy coat C-PRF to prepare the Alb-PRF [20].

Our observation that PPP holds a TGF-β activity was originally unexpected. We can only speculate about the source of TGF-β being responsible for changing the expression of IL11 and NOX4 in the gingival fibroblasts. Basal TGF-β levels in blood plasma are low at around 5 ng/mL and it is the coagulation process that liberates the growth factor from platelet granules and, presumably, also other cells [35]. It is probably the activation of platelets during the blood draining and centrifugation even with hydrophobic plastic tubes that releases TGF-β into the PPP fraction. Support for this assumption comes from platelet glycoprotein IIb positive (CD41^+^)-cell imaging, suggesting that platelet distribution in solid PRF is not gravity-dependent [36]. Further support for this claim came from studies where PPP increased the expression of typical TGF-β target genes, such as connective tissue growth factor (CTGF), in tendinopathic cells [37] and hypertrophic scar dermal fibroblasts [38], considering that the primary effects of TGF-β are at least partially mediated via this growth factor [39]. Similar to CTGF, it is IL11 and NOX4 that are regulated by PPP lysates that mediate the effects of TGF-β on cardiovascular and liver fibrosis [29,30], and acute kidney injury and pulmonary fibrosis [31,34], respectively. Thus, IL11 and NOX4 are not only sensitive TGF-β target genes in vitro as they mediate at least part of the TGF-β activity in vivo.

Our finding that the PPP contains a TGF-β activity should also become important in the high-speed protocols used to prepare solid L-PRF [10]. In L-PRF, platelets and leucocytes accumulate in the buffy coat layer [10]—but presumably, also the PPP part of the solid L-PRF holds some TGF-β activity. We are currently evaluating the TGF-β activity in the fractions of solid PRF. Our findings are consistent with other reports that even centrifugation for 15 min at 10,000 g cannot fully remove platelets from anticoagulated plasma; a median of 2 × 10^3^ platelets/µL remained in the plasma [40]. Moreover, centrifugation activates platelets; the percentage of activated platelets in anti-coagulated whole blood prior to centrifugation is 4–9% and increases to around 50% based on the expression of P-selectin [40]. In addition, the analysis of the PPP fractions using centrifugation 12 min at 700 g [41] and 8 min at 3000 g [42] showed the presence of platelets. Again, platelet distribution in solid PRF was not gravity-dependent based on CD41 stainings [36]. This accumulating evidence suggests that the PPP fraction should not be neglected as a source of TGF-β activity. Altogether in support of the proposed original protocol [20], when preparing heated PPP with denaturated albumin, mixing it back with the concentrated liquid C-PRF is necessary to reconstitute the TGF-β activity in Alb-PRF.

## 4. Materials and Methods

### 4.1. Cell Culture

An approval for collecting human gingiva was obtained from the Ethics Committee of the Medical University of Vienna (EK NR 631/2007), and patients signed informed consent. Three different strains of fibroblasts were prepared from explant cultures. Gingival fibroblasts were expanded in growth medium containing penicillin, streptomycin (Sigma Aldrich, St. Louis, MO, USA) and 10% fetal bovine serum (Bio&Sell GmbH, Nuremberg, Germany). Fibroblasts at 30,000 cells/cm^2^ were exposed to lysates of unheated and heated PPP (Alb-gel), buffy coat C-PRF, and the red blood clot in serum-free medium for 24 h at 37 °C, 5% CO_2_, and 95% humidity. TGF-β receptor type I kinase was blocked with 10 µM SB431542 (Calbiochem, Merck, Billerica, MA, USA).

### 4.2. Preparation of PPP, Buffy Coat and Red Clot

Volunteers signed informed consent and the ethics committee of the Medical University of Vienna (1644/2018) approved the preparation of PRF. For preparing PRF gels [20], venous blood was collected from healthy volunteers, three females and three males from 23 to 35 years, in plastic tubes (“No Additive“, Greiner Bio-One GmbH, Kremsmünster, Austria) and centrifuged at 700 g for 8 min (swing-out rotor; Z 306 Hermle, Universal Centrifuge, Wehingen, Germany). The uppermost 2 mL PPP and the 1 mL buffy coat, as well as 1 mL erythrocyte fraction were collected. To generate PPP gels [20], PPP was immediately heated at 75 °C for 10 min (Eppendorf, Thermomixer F1.5, Hamburg, Germany) before being placed on ice [12]. Each blood fraction was subjected to dual freeze thawing followed by sonication (Sonopuls 2000.2, Bandelin electronic, Berlin, Germany). After centrifugation (Eppendorf, Hamburg, Germany) at 15,000 g for 10 min, 1 mL of the lysate was mixed with 0.5 mL serum-free medium and stored at −20 °C for not longer than one month. Once cells were ready for being stimulated, the fractions were thawed and cells were exposed as indicated above.

### 4.3. Reverse Transcription Quantitative Real-Time PCR (RT-qPCR) and Immunoassay

For RT-qPCR [43], total RNA was prepared with the ExtractMe total RNA kit (Blirt S.A., Gdańsk, Poland) followed by reverse transcription (SensiFAST^TM^ cDNA kit, Bioline, London, UK) and polymerase chain reaction (SensiFAST^TM^ SYBR ROX Kit, Bioline). Primer sequences were hGAPDH-F: aag cca cat cgc tca gac ac, hGAPDH-R: gcc caa tac gac caa atc c.; hNOX4a-F tct tgg ctt acc tcc gag ga, hNOX4a-R: ctc ctg gtt ctc ctg ctt gg and hIL11 (qHsaCEP0049951; Bio-Rad Laboratories, Inc., Hercules, CA, USA). Amplification was performed with the CFX Connect^TM^ Real-Time PCR Detection System (Bio-Rad Laboratories, CA, USA). The expression levels were calculated by normalizing to the housekeeping gene GAPDH using the ΔΔCt method. The immunoassay for human IL11 (DY218, R&D Systems, Minneapolis, MN, USA) was performed with the supernatant of gingival fibroblasts exposed to lysates of PPP, heated PPP (Alb-gel), buffy coat (BC) and red clot (RC) after 24 h. Immunoassay for human active TGF-β1 (DY218, R&D Systems) was conducted with lysates of the blood fractions.

### 4.4. Immunofluorescence

Gingival fibroblasts were plated onto Millicell^®^ EZ slides (Merck KGaA, Darmstadt, Germany) at 15,000 cells/cm^2^. Cells were serum-starved before being exposed to 30% of lysates of PPP, heated PPP, buffy coat C-PRF and 5% of red clot for 30 min. The cells were immediately fixed with 4% formaldehyde, blocked with 1% bovine serum albumin and permeabilized with 0.3 % Triton. We used rabbit anti-smad2/3 antibody (D7G7 XP^®^) at 4 °C overnight. Detection was done with the goat anti-rabbit Alexa 488 secondary antibody (CS-4412, 1:1000, Cell Signaling Technology, Danvers, MA, USA). Images were captured with a fluorescent microscope (Oxion fluorescence, Euromex, Arnheim, The Netherlands).

### 4.5. Western Blot

Gingival fibroblasts seeded at 30,000 cells/cm^2^ were serum-starved overnight. Cells were exposed for 30 min to 30% of lysates prepared from PPP, heated PPP (Alb-gel), buffy coat C-PRF, and 5% lysates of the red clot. Protein extracts in SDS buffer containing protease and phosphatase inhibitors (cOmplete ULTRA Tablets and PhosSTOP; Roche, Mannheim, Germany) were separated by SDS-PAGE and transferred onto nitrocellulose membranes (Whatman, General Electric Company, Fairfield, CT, USA). The binding of rabbit p-Smad3 antibody (phospho S423 + S425, [EP823Y], Abcam, Cambridge, UK) was detected with an antibody labelled with peroxidase (CS-7074, anti-rabbit IgG, Cell Signaling Technology), respectively. Peroxidase was visualized with Clarity Western ECL Substrate (Bio-Rad Laboratories, Inc., Hercules, CA, USA) and signals detected with the ChemiDoc imaging system (Bio-Rad Laboratories).

### 4.6. Statistical Analysis

All experiments were performed three to five times. Bars show the mean and standard deviation of the cumulative data from the means of independent experiments. Statistical analysis of the IL11 and NOX4 expression and immunoassay was performed with ANOVA and post hoc Fischer’s LSD test for multiple comparison comparing all groups with the Alb-gel group. The impact of SB431542 on gene expression changes was based on Mann-Whitney U test. The least significant dose of PPP and BC in IL11 expression was determined with an ANOVA and post hoc Fischer’s LSD test for multiple comparison to compare all groups with each other. Analyses were performed using Prism v8 (GraphPad Software, La Jolla, CA, USA). Significance was set at *p*  <  0.05.
